# A Tyrosine Residue on the TSH Receptor Stabilizes Multimer Formation

**DOI:** 10.1371/journal.pone.0009449

**Published:** 2010-02-26

**Authors:** Rauf Latif, Krzysztof Michalek, Syed Ahmed Morshed, Terry F. Davies

**Affiliations:** 1 Thyroid Research Unit, James J. Peters VA Medical Center, Mount Sinai School of Medicine, New York, New York, United States of America; 2 Department of Endocrinology, Metabolism and Internal Diseases, Poznan University of Medical Sciences, Poznan, Poland; Albert Einstein Institute for Research and Education, Brazil

## Abstract

**Background:**

The thyrotropin stimulating hormone receptor (TSHR) is a G protein coupled receptor (GPCR) with a large ectodomain. The ligand, TSH, acting via this receptor regulates thyroid growth and thyroid hormone production and secretion. The TSH receptor (TSHR) undergoes complex post –translational modifications including intramolecular cleavage and receptor multimerization. Since monomeric and multimeric receptors coexist in cells, understanding the functional role of just the TSHR multimers is difficult. Therefore, to help understand the physiological significance of receptor multimerization, it will be necessary to abrogate multimer formation, which requires identifying the ectodomain and endodomain interaction sites on the TSHR. Here, we have examined the contribution of the ectodomain to constitutive multimerization of the TSHR and determined the possible residue(s) that may be involved in this interaction.

**Methodology/Principal Findings:**

We studied ectodomain multimer formation by expressing the extracellular domain of the TSHR linked to a glycophosphotidyl (GPI) anchor in both stable and transient expression systems. Using co-immunoprecipitation and FRET of tagged receptors, we established that the TSH receptor ectodomain was capable of multimerization even when totally devoid of the transmembrane domain. Further, we studied the effect of two residues that likely made critical contact points in this interaction. We showed that a conserved tyrosine residue (Y116) on the convex surface of the LRR3 was a critical residue in ectodomain multimer formation since mutation of this residue to serine totally abrogated ectodomain multimers. This abrogation was not seen with the mutation of cysteine 176 on the inner side of the LRR5, demonstrating that inter-receptor disulfide bonding was not involved in ectodomain multimer formation. Additionally, the Y116 mutation in the intact wild type receptor enhanced receptor degradation.

**Conclusions/Significance:**

These data establish the TSH receptor ectodomain as one site of multimerization, independent of the transmembrane region, and that this interaction was primarily via a conserved tyrosine residue in LRR3.

## Introduction

The thyroid stimulating hormone receptor (TSHR), a typical 7-transmembrane GPCR, on the surface of thyrocytes, is the master regulator of thyroid growth and development. TSH acting via TSHR regulates thyroid hormone production and secretion. In addition TSHR is also a major autoantigen for autoimmune diseases of the thyroid gland [1]–[4]. The TSHR consists of a large extracellular ectodomain of 415 residues inclusive of a signal peptide of 21aa. The 10 leucine rich repeat regions (LRR) on the ectodomain is the main region for TSH and TSHR antibody binding. The membrane associated intracellular carboxyl terminal endodomain of 384aa encoded by the 10th exon consists of the 7 transmembrane domains and a short cytoplasmic tail [5]. Unlike other glycoprotein hormone receptors, this receptor has a unique 50 amino acid region (residues 316–366) on its extracellular domain which undergoes proteolytic degradation[6] by an unidentified matrix metalloprotease resulting in the cleavage of the receptor into α (or A) and β (or B) subunits held together by disulfide bonds [7]–[9].

It is well established that GPCRs may exist as dimers and higher order complexes such as oligomers/multimers [10]–[12]. The TSHR, in addition to undergoing intramolecular cleavage, also exists as dimers and higher order forms both in native tissue and transfected cells [13]–[15]. Our laboratory not only showed the existence of these higher order complexes in native porcine membranes and in heterologous cells [13], [14] but also observed that these di(multi)meric complexes were regulated by TSH ligand [16], a phenomenon that appeared to be exaggerated for TSHRs residing in “lipid rafts” [17] - cholesterol and sphingolipid rich domains on the plasma membrane.

TSHR homodimerization, confirmed by Foster Resonance Energy Transfer (FRET) and Bioluminescence Resonance Energy Transfer (BRET), has been shown to play a functional role in negative cooperativity by allosteric modulation [15]. Although the phenomenon of negative cooperativity with TSHRs was well known earlier [18], [19], the phenomenon can be explained by the observation that the TSHR is capable of existing in dimeric and multimeric forms [15]. Another functional role for TSHR dimerization has been in the mechanism of TSH resistance seen in congenital hypothyroidism. Trafficking of the wild type receptor to the cell surface was inhibited when co-expressed with a mutant TSHR [20], [21]. We have also observed that cross-linking the TSHRs, using receptor specific monoclonal antibodies, appeared to reduce intra-molecular cleavage of the receptors which in turn prolonged their cell surface expression [22]. This observation has possible implications for the prolonged stimulation of the TSHR autoantigen [22]. Furthermore, although multimerization has not been proven to be directly involved in receptor signaling, we have observed that TSHR multimers are enriched in “lipid rafts” coexisting with G proteins and monomers [17]. These observations suggest that it may take both monomeric and multimeric forms of receptor to coexist for the normal physiological functions of thyrocytes as suggested for other GPCRs [23].Therefore the role of higher order forms of the TSHR in physiology and pathology of the thyroid remains open to investigation.

Co-existence of monomeric and di (oligo) meric forms of TSHRs on the thyroid cell surface makes it difficult to delineate the precise role of each form in signal transduction or receptor trafficking. Furthermore, cleaved and uncleaved forms of the receptor are capable of aberrant homodimerization [24] and influence receptor signaling adding to the complex nature of receptor interactions. Therefore, to study the impact of these higher order receptors, it will be necessary to first try and inhibit constitutive dimerization/multimerization and then examine the physiological consequences of such inhibition. We have, therefore, begun to define the dimerization interfaces by determining the contact points on the large ectodomain. The Follicle Stimulating Hormone (FSH) receptor, which has a similarly large ectodomain, when devoid of the transmembrane domain, was still capable of dimerization [25] when the first 268 amino acids of the ectodomain were crystallized in the presence of FSH. Following the FSH crystal structure, the TSH receptor extracelluar domain (TSHR260) complexed with a TSHR antibody (M22) Fab has been solved to a resolution of 2.55A [26]. Hence, we hypothesized that the TSHR extracellular domain may harbor dimerization motifs and in this report we confirm that 1) The TSHR ectodomain is indeed capable of forming higher order complexes independent of its transmembrane domain, 2) We defined the major contact point for this interaction and 3) We analyzed the role of the contact residues in receptor stability.

## Results

### Surface Expression of GPI-TSHR-ECD Constructs

**Figure 1:**
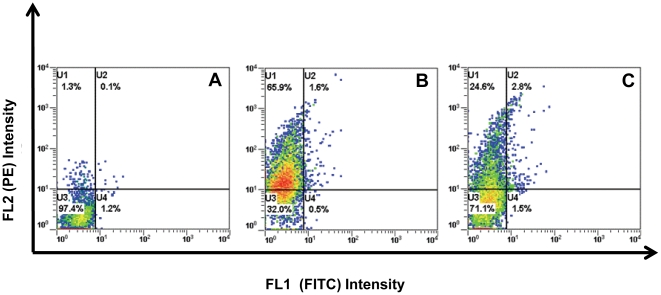
Expression profile of HA and Myc tagged GPI-TSHR-ecd receptors: HEK293 cells were transiently transfected with 1 ug of HA or Myc-GPI-TSHR-ecd plasmid cDNA and the cells were stained 48 hrs post-transfection with anti-HA or anti-Myc (1 ug/10^6^cells) antibody. This was followed by anti-mouse IgG Fab' conjugated to phycoerthrin (PE) as secondary antibody. Density analysis for receptor expression under FL2 (PE) and FL1 (FITC) was performed using the Beckman Lab Quanta SC flowcytometer. **Panel A**) Controls cells transfected with vector only and stained with primary and secondary antibodies. **Panel B**) HA-GPI-TSHR-ecd transfected cells stained with anti-HA (66% positivity). **Panel C**) Myc-GPI-TSHR-ecd transfected cells stained with anti-myc (25% positivity). Since tags are the N-terminus of the receptor ectodomain, unfixed cells were used for this staining.

To study the role of the extracellular portion of the TSHR in constitutive dimerization/multimerization, we used the TSHR ectodomain (1-412aa) linked to a GPI anchor. The cDNA construct was stably expressed in Chinese Hamster Ovary (CHO) cells (CHO-GPI-TSHR-ecd) and also transiently expressed in Human Embryonic Kidney (HEK) 293 cells. We first tagged the amino terminus of the GPI-TSH-ecd receptors (with HA or Myc) and assessed their expression on the cell surface of transiently transfected HEK293 cells. This was confirmed by staining unfixed cells using anti-HA or anti-Myc monoclonal antibodies by flow cytometry (Figure 1). These studies demonstrated a good degree of expression with a Mean Fluorescence Intensity (MFI) of >250AU for each of these tagged constructs with 65% of cells positive for HA and 25% of cells positive for Myc expression

### Existence of Higher Order Forms of TSHR Ectodomain

**Figure 2:**
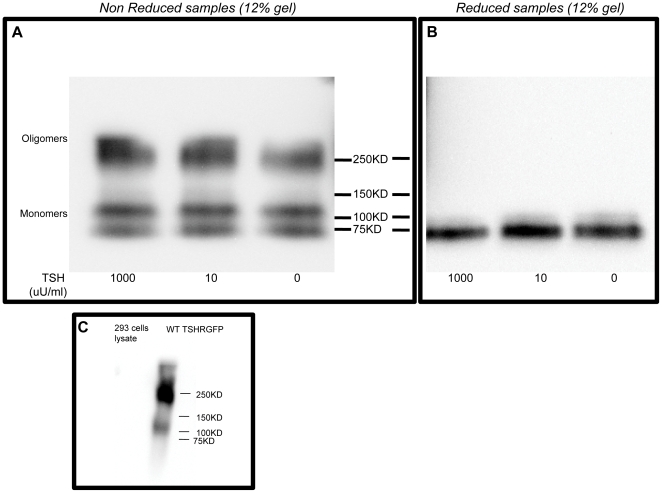
Immunoblot of total membranes under reduced and non-reduced conditions from stably transfected cells: CHO cells stably expressing GPI-TSHR-ecd were treated with 0, 10 and 1000 µU/ml of bovine TSH for 1 hr at 37°C. Membranes prepared from untreated and treated cells was divided into 30 ug/lane and treated with 5X sample buffer containing reducing agent (+DTT: 100 mM final) and non-reducing agent (-DTT) and separated on a 12% SDS-PAGE. Proteins were transferred onto PVDF and probed by 2 ug/ml TSHR antibody M4 (epitope 322–341) followed by detection with anti-mouse HRP (1:20,000) and developed using pico SuperSignal ECL (Pierce Ltd). **Panel A**) Shows the non-reduced untreated and treated samples. Oligomeric forms of 200–250 KD were observed in addition to the mature 100KD and less mature 75 KD monomeric forms GPI linked TSHRs. **Panel B**) Untreated and treated samples after reduction with DTT for 45 minutes at 50°C showing no higher order forms of the TSHR-ecd indicating the instability of the TSHR-ecd higher order forms. This is a representative blot from two experiments performed under identical conditions. **Panel C**) the specificity of the antibody M4 used for immunoblotting throughout the study is indicated in this blot. Lysates from untransfected HEK 293 cells and TSHRGFP transfected wild type cells were resolved on SDS-PAGE and immunblotted with 2 ug/ml of M4 antibody. M4 did not have any reactivity with untransfected lysate as opposed to TSHR transfected lysate where it detected the monomeric and multimeric forms of the receptor.

**Figure 3:**
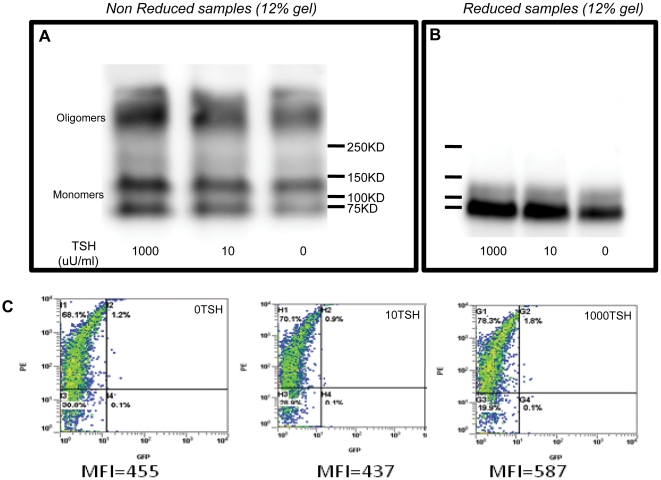
Immunoblot of total membranes under reduced and non-reduced conditions from transiently transfected HEK cells: HEK 293 cells transiently transfected with HA-GPI-TSHR-ecd were treated with 0, 10 and 1000 µU/ml of bovine TSH for 1 hr at 37°C. Total membranes prepared from untreated and treated cells were divided into 30 µg/lane and treated with 5X sample buffer containing reducing agent (+DTT:100 mM DTT final) and non-reducing agent (-DTT) and the proteins separated by 12% SDS-PAGE. The blots were probed further using 2 µg/ml TSHR antibody M4 (epitope 322–341) followed by detection with anti-mouse HRP (1:20,000) and developed using pico SuperSignal ECL (Pierce Ltd). **Panel A**) Shows the untreated and TSH treated samples under non-reduced conditions. **Panel B**) The same set of samples as in Panel A after reduction with DTT for 45minutes at 50°C. Results are similar to that observed with stably expressed cells in Figure 2. **Panel C**) Flow cytometric data of untreated versus treated cells. TSH treatment did not alter the expression profile of HA in these HA-GPI-TSHR-ecd transiently transfected cells. The expression levels of the tagged receptor are indicated by their mean fluorescence intensity (MFI).

We next analyzed total membranes prepared from CHO-GPI-TSHR-ecd [16] (G13 cells) with and without TSH exposure and under non-reducing and reducing conditions. The blots when probed with a TSHR antibody M4 (RSR 4: recognizing residue 322–341) [27], revealed the presence of higher order forms in addition to the fully mature monomeric form of GPI: TSHR–Ecd consisting of a doublet comprising of a 100KD form and less mature molecular species of 74KD as observed by others [28]. Distinct bands of 250KD corresponding to higher order forms of the ectodomain was observed in untreated and TSH treated samples under non-reduced conditions (Figure 2A). However, reduction of these samples with 100 mM DTT for 45 minutes at 50°C, caused the multimeric forms as well as the 100KD form to be reduced into less mature form of the TSHR ectodomain (Figure 2B). This was observed even in transiently transfected HEK 293 cells as shown in Figure 3A and 3B. Previously we had observed that TSH treatment of full length receptors stably expressed in CHO cells caused a marked decrease in multimeric forms both in total membranes and lipid raft preparations under non-reducing conditions [17]. This lack of effect of TSH on ectodomain expression was confirmed by flow cytometry (Figure 3C).

### Homo-Interaction of TSHR Ectodomains and the Effect of Tyrosine (Y116) and Cysteine (C176) Mutations

Molecular modeling and bioinformatic approaches have clearly shown that transmembrane domains are the potential regions of dimerization or multimerization of several GPCRS [29], [30] although the role of the ectodomain in this process of receptor-receptor interaction is still unclear. To extend our observations on multimers observed in non-reduced conditions and to study the effect of mutations on the interaction of receptors, we used co-immunoprecipitation of the tagged TSHR ectodomain receptor constructs.

**Figure 4:**
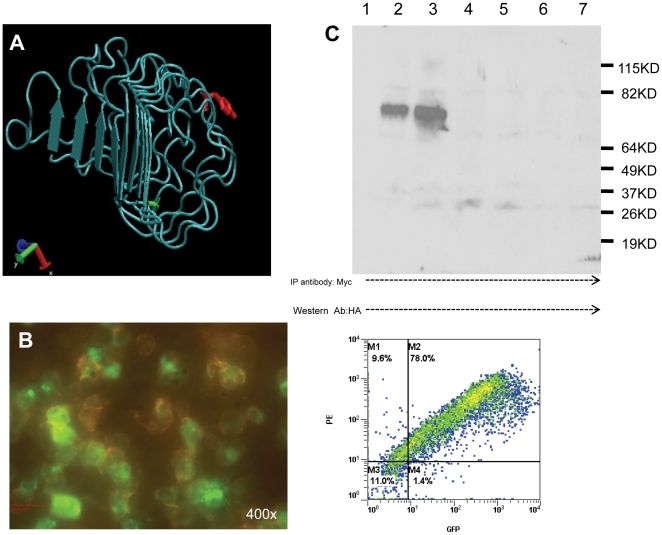
Co-immunoprecipitation of HA and Myc tagged TSHR ectodomain and its mutants: **A**) the position of Y116 and C176 are indicated on the ECD structure. Y116 is on the outer convex surface of the LRR3 (red) and C176 (green) is on the concave inner surface of the LRR5. The coordinates for the ectodomain structure of TSH receptor was obtained from the GRIS database [43] and this structure was examined in the open source molecular graphics program VMD (www.ks.uiuc.edu/Research/vmd) to mark the position of Y116 and C176 on the ECD structure. **B**) Expression of the Y116 on HEK 293 cells is indicated both microscopically and quantitatively by flow cytometry. The yellow staining observed on the cell surface was due to double staining of GFP (on the C-terminus of construct) and TSHR antibody (RSR1 or M1 residue 381–385) labeled with secondary antibody conjugated to texas red. The FACS profiles on right shows that 78% of these cells to be double positive. The C176 construct expressed on HEK 293 had similar levels of expression (data not shown). **C**) as described above, constructs carrying the Y116 or C176 mutations (in the HA-GPI-TSHR-ecd) were co-transfected with Myc-GPI-TSHR-ecd. Membranes prepared from single and double transfected cells were immunoprecipitated using anti-Myc monoclonal antibody 9E10 (2 µg/ml final). The PVDF transferred immune complex was probed with anti-HA (1 µg/ml) and developed further with secondary antibody conjugated to HRP. **Lane 1**) Membranes from single transfected cells (*HA-GPI-TSHR-ecd only*) and subjected to identical co-immunoprecipitation conditions. **Lane 2**) Membranes from double transfected cells (*HA-GPI-TSHR-ecd+Myc-GPI-TSHR-ecd*). **Lane3**) Membranes from double transfected cells (*C176Δ HA-GPI-TSHR-ecd+Myc GP-TSHR-ecd*). **Lane 4**) Membranes from double transfected cells (*Y116 Δ HA-GPI-TSHR-ecd + Myc-GPI-TSHR-ecd*). **Lane 5**) Membranes from single transfected cells (*C176Δ HA-GPI-TSHR-ECD only*). **Lane 6**) Membranes from single transfected cells (*Y116 mutant HA-GPI-TSHR-ecd only*) and **Lane 7**) Membranes from untransfected HEK293 cells. Only monomeric forms are seen with immunoprecipitation and reduction in lanes 2 and 3. Mutation of Y116 caused loss of receptor interaction and higher order forms and thus no immunoprecipitation of TSHR-ecd (lane 4).

We mutated residues corresponding to C176 and glycoprotein receptor conserved Y116on the ectodomain of the HA-GPI-TSHR-ecd construct into alanine and serine respectively. The location of residues Y116 and C176 are indicated on the structural model of the receptor ectodomain in Figure 4A. The mutated constructs were sequence verified prior to the interaction studies. The expression of these mutant receptor forms was also verified on unfixed and fixed cells by flow cytometry using anti-HA and TSHR specific monoclonal antibody M1 (recognizing residues 381–385) as described above (Figure 4B). Furthermore, membranes prepared from single and double transfected cells were immunoprecipitated with anti-Myc and the immunoprecipitates were analyzed with anti-HA. Ectodomain-ectodomain interaction was confirmed when we observed that anti-myc pulled down the less mature 75KD receptor complex when probed with anti-HA in membrane preparations from HA-GPI-TSHR-ecd+Myc-GPI-TSHR-ecd co-transfected cells. In contrast, no immunoprecipitates were observed from membranes of single transfected HA-GPI-TSHR-ecd cells (Figure 4C, Lanes 1 & 2). However, immunoprecipitation of membranes of HA-GPI-TSHR-ecd with Y116 mutation + Myc-GPI-TSHR-ecd also failed to result in the 75KD protein (Lane 4) in contrast to 75KD protein band observed in membranes from C176 mutated HA-GPI-TSHR-ecd + MycGPI-TSHR-ecd transfected cells (Lane 3). Other controls such as the Y116 mutated HA-GPI-TSHR-ecd single transfected cells and untransfected membranes did not yield any protein bands corresponding to these receptor sizes.

These data further confirmed the interaction of TSHR ectodomains. The results also demonstrated Y116 on the outer alpha helix loop of LLR3 as a critical interacting partner since mutation of this residue to serine abrogated ectodomain-ectodomain interactions.

### Homo-Interaction of TSHR Ectodomain in Live Cells

**Figure 5:**
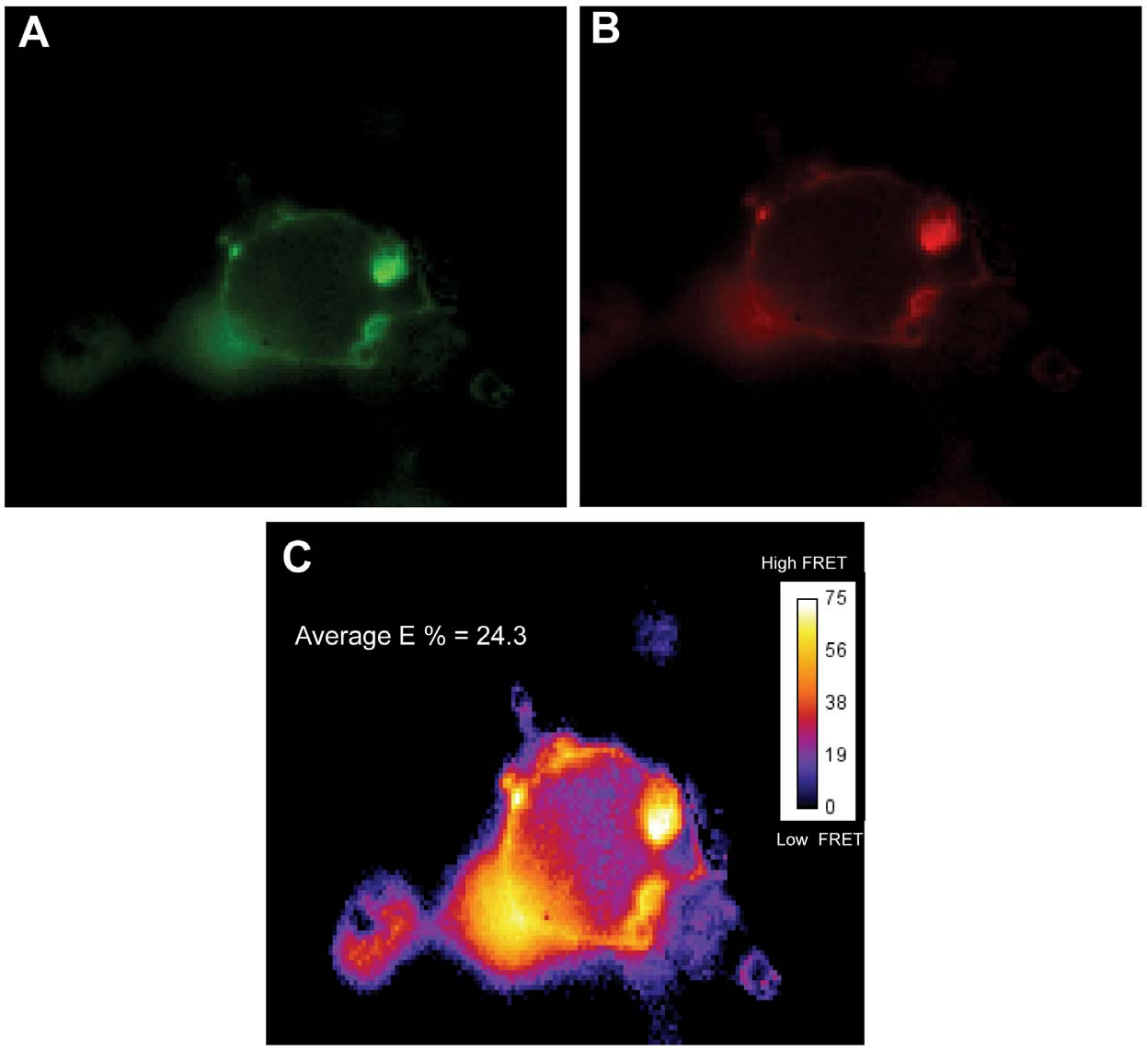
FRET on HA and Myc tagged ectodomain constructs in HEK 293 cells. HA-GPI-TSHR-ecd and myc-GPI-TSHR-ecd were co-transfected into HEK 293 cells and stained with anti-HA FITC and anti-Myc Cy3 labeled antibody and imaged for energy transfer between FITC (donor) and Cy3 (acceptor) fluorophores. As described in Materials and Methods single transfected cells labeled with anti-HA-FITC and anti-Myc-Cy3 alone were the endogenous control for lack of FRET and for correction of donor and acceptor spectral bleed through. **Panel A** shows the staining of GPI-TSHR-ecd by anti HA FITC. **Panel B** shows the same cell stained with anti Myc Cy3. **Panel C** is the pseudo colored FRET energy level image obtained from the analysis of the double transfected cells by using the pFRET software. The calibration bar indicates the FRET energy t level in the cell. Energy transfer was in the range of 8–10% on the surface of the cells compared to controls (TSHRGFP +TSHRmyc labeled with Cy3) which showed energy transfer of 24%.

The biochemical evidence of ectodomain interaction observed above was confirmed in live cells. HEK 293 cells transfected with HA-GPI-TSHR-ecd + Myc-GPI-TSHR-ecd were used for FRET studies. FRET was performed using anti-HA FITC as the donor molecule and anti-Myc Cy3 as acceptor molecule. Cells labeled with donor alone and acceptor alone were used as endogenous controls for this FRET studies. In each experiment, data acquired from five single transfected and double transfected cells were analyzed for FRET efficiency after removal of background and spectral bleed-through by using the pFRET software [31]. We were able to observe FRET with an efficiency of 8–10% in HA-GPI-TSHR-ecd + Myc-GPITSHR-ecd transfected cells which was lower than the control cells with full-length receptors tagged at their carboxyl end with GFP as donor and Myc antibody labeled with Cy3 as an acceptor (FRET efficiency of 20–25%, Figure 5). There was no FRET in single transfected cells. The FRET data on these N-terminus tagged GPI-TSHR-ecd cells further confirmed the interaction of ectodomains observed with immunoprecipitation.

### Y116 Mutation on Wild Type TSHR Leads to Receptor Degradation

**Figure 6:**
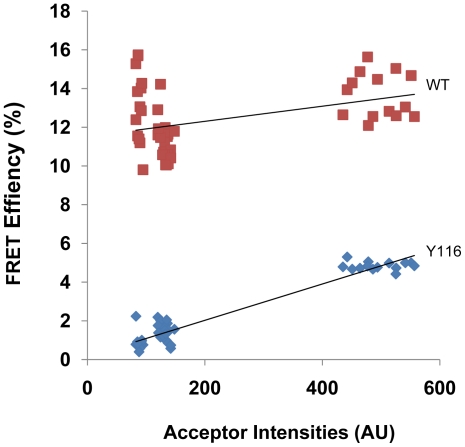
Effect of Y116 mutation on receptor. FRET efficiency of Y116 and full length TSHR are indicated at low and high acceptor values. Y116 shows decreased FRET efficiency compared to the WT type cells.

**Figure 7:**
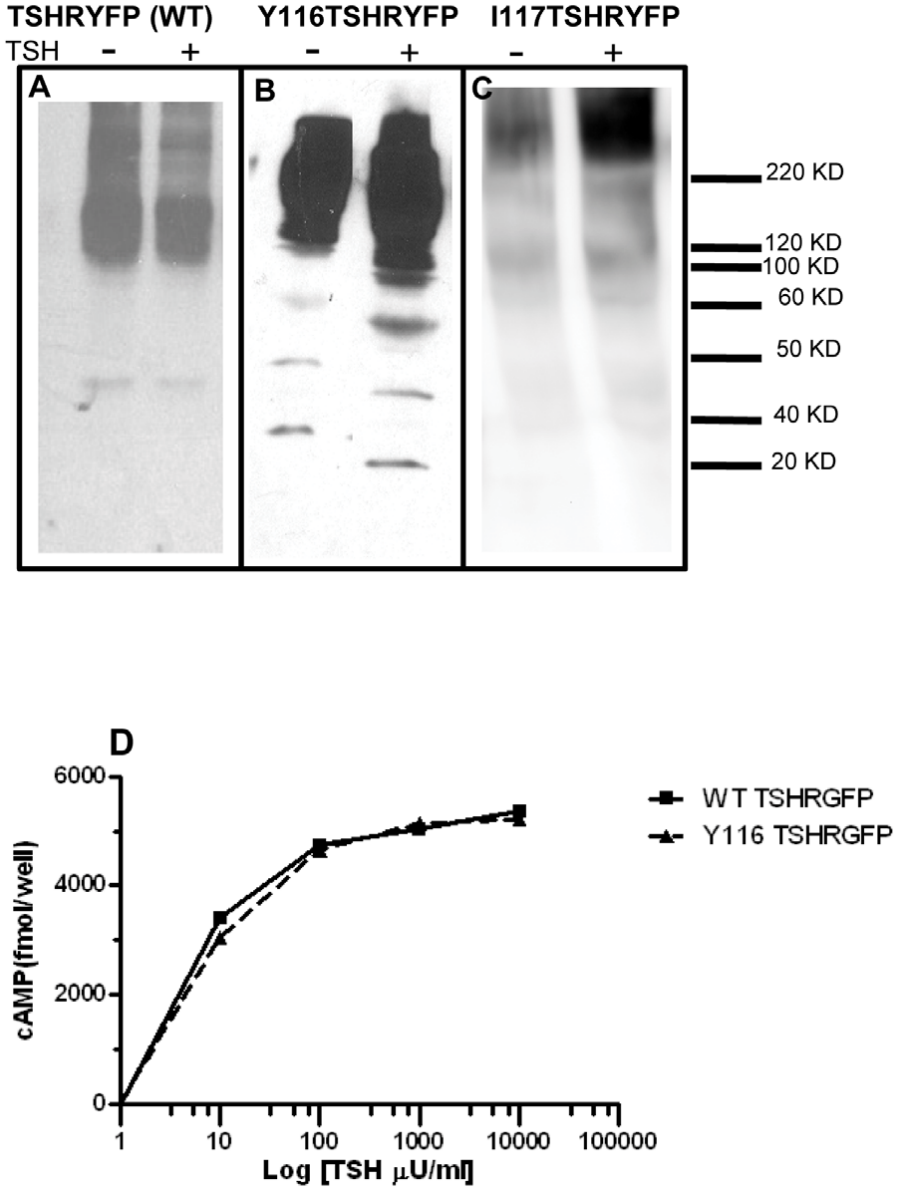
Effect of Y116 mutation on receptor degradation. **Panels (A, B and C)** Solubilized receptors separated under non-reduced conditions from TSHRYFP (WT), Y116 and I117 mutated receptor transfected cells at 0 and 30 minutes after 1000 µU/ml of bovine TSH. The Y116 mutated receptors showed enhanced degraded forms of 60KD to 20KD which was not observed with the wild type receptor nor I117 mutated receptors. **Panel D**: HEK 293 cells transfected with the WT receptor (TSHRYFP) and Y116 mutated receptor where stimulated with varying doses of bovine TSH. A dose-dependent cAMP response of the two receptors is shown in the graph. Y116 mutation on ectodomain had no effect on the response of receptors to TSH.

To study if the Y116 mutation had any effect on receptor degradation in the context of the transmembrane region, we mutated Y116 to serine on the full length TSHR_GFP_. FRET efficiency between the Y116 mutated TSHR_GFP_ construct and the Myc labelled non-mutated TSHR_myc_ tagged full-length (WT) receptor was observed to be lower than the control (Figure 6) [16]. We further studied the structural forms of the receptor in total membranes under non-reduced condition to examine if the decrease in FRET was due to receptor degradation. Y116 mutated receptor preparations showed lower molecular weight degraded forms (Figure 7B) which increased with time of TSH treatment. No degraded forms were observed in the non mutated WT receptor membrane preparations even after 30 minutes of TSH treatment (Figure 7A). These data suggested a role for Y116 in receptor stability. A control mutation, isoleucine 117 (I117), in the outer loop of LLR3 adjacent to Y116 also did not lead to any increased constitutive receptor degradation. (Figure 7C).The cAMP signaling capability of Y116 receptor, however, was not affected by the enhanced degradation of the receptor forms (Figure 7D) suggesting ectodomain degradation was post-signaling.

## Discussion

Glycoprotein hormone receptors such as the LHR, FSHR and TSHR belonging to Class A receptors exist as dimeric and higher order forms in native and transfected cells [13]–[15], [32]–[34] although the physiological relevance of these receptor forms is still under investigation. Therefore, to understand the exact biological role of dimers and/or higher order forms of receptors it will be necessary to generate GPCRs that are expressed only as monomeric or multimeric receptor forms in model cell systems. This requires mapping the several dimerization/multimerization interfaces of the receptors and to mutate the critical interface residues without compromising the function of these receptors. While we, and others, have shown previously that the transmembrane region of the TSHR is the major region of dimer or oligomer formation [14], [15], the potential role of the large ectodomain (412 aa in size) in receptor- receptor interaction and stabilizing such receptor forms has not been delineated. The present study, therefore, was designed primarily to understand this role of the ectodomain on receptor-receptor interaction.

We expressed untagged and tagged TSHR ectodomains (residues 1–412) in mammalian cells and found higher order forms of the receptor under non-reduced conditions. That these forms resulted secondary to interaction of the ectodomain receptors in vivo was confirmed by the FRET analyses. Hence, the data demonstrated that TSHR ectodomains were capable of forming higher order forms in the absence of the transmembrane domain. However, these higher order forms of ectodomain were unstable on mild reduction with DTT. While it is not uncommon for DTT to have nonspecific effects on the dissociation of whole receptor complexes as observed with the P2Y12 and β2AR GPCRs [35], [36], the reduction of higher complexes by DTT suggested that ectodomain oligomer formation may be mediated, in part, by disulfide bond formation via the several cysteine residues found on the ectodomain of the TSHR [1], [6]. It has been established that 10 of the 11 cysteine residues in the ectodomain of the TSHR pair with each other resulting in 3 cysteine boxes [1], [6]. This arrangement leaves C176, located on the inner side of the concave surface of the leucine rich region (LRR), as the only unpaired cysteine. Being the only unpaired cysteine, we speculated that cysteine 176 (C176) may be a potential candidate for the formation of intermolecular disulfide bond formation. This justified our choice of cysteine 176 as a possible residue to mutate. Since disulfide bond formation is not the only mechanism of oligomer formation in GPCRs [10], [37] we also examined tyrosine 116 located on the outer loop of LRR 3 based on the reported crystal structure of the FSH-FSHR_HB_ existing as a biologically plausible dimer contact point [25]. This study of the FSHR showed that Y116 was conserved among LHR, FSHR and TSHRs with a 0.1 fractional solvent accessibility score which suggested it may be a critical residue in the dimer interface. We had earlier established, by biochemical and biophysical approaches, that the full length TSHR is able to form dimeric and higher order forms in native and transfected cells [13], [16], [17]. These di-(oligo)meric forms of TSHR resided on the surface of the cells as shown by FRET using reporter tagged receptors and HFRET using donor and acceptor labeled antibodies [15], [16]. Studies on oligomer formation in GPCRs has largely focused on the involvement of either full-length receptors or the transmembrane regions with very few reports of extracellular amino-terminal domain involvement [37]–[39]. The present study clearly demonstrated that TSHR ectodomains, independent of their transmembrane regions, could form higher order oligomers. Using differentially tagged GPI-TSHR-ecds, we succeeded in confirming this phenomenon using co-immunoprecipitation and FRET on live cells. Although the FRET efficiency in ectodomain expressing cells was lower compared to the data obtained with full-length TSHRs this was most likely due to the combined use of two labeled antibodies which can result in unfavorable dipole orientations.

To study the effect of the two potentially important residues in ectodomain multimers, we mutated C176 in LRR5 and Y116 in LRR3 to serine in the tagged constructs. The two mutated receptors showed no obvious trafficking abnormality and were detected on surfaces of unfixed cells. Furthermore, co-immunoprecipitation of membranes prepared from double transfected cells showed that mutation of C176 did not abrogate the receptor-receptor interaction. However, mutation of Y116 to serine resulted in the loss of receptor-receptor interaction leading to no immunoprecipitation. It has been recently shown that the FSHR ectodomains in the FSH-FSHR_HB_ crystal structure also interact to constitute a dimer [25]. According to this study the dimer interface involved three stranded β-sheets (LRR 2-4) on the outer surface of the FSHR_HB_ and Y116 contributed to a strong hydrophobic interaction mediating 21 of the 24 direct carbon-carbon contacts [25]. Since Y116 is completely conserved among the glycoprotein hormone receptors, it was important to examine this residue in the context of receptors expressed on the cell surface in the absence of the transmembrane domain. Looking at the structural details of the TSHR ectodomain in the GRIS database (http://gris.ulb.ac.be) we found that Y116 makes a hydrogen bond with residue Asp 65 (3061) and several hydrophobic contacts although there are no salt bridges reported for this residue further suggesting the interactions to be weak. However, a follow up study on the structural determinants of FSHR dimerization showed that mutation of Tyr110 to alanine did not inhibit dimerization [40]. Although the FSHR and the TSHR belong to the same family of glycoprotein receptors and have presumably similar ligand binding orientation [26] the discrepancy with the study of Guan et al [40] can only be due to the following differences 1) We employed ECD constructs tagged at the amino terminus with HA and myc to do our co-IP and FRET studies rather than transmembrane linkers such as LRP6 and CD8 tagged at their cytoplasmic end with donor acceptor pairs. The short GPI anchor to the ectodomain may have provided it the ability to orient more freely on the plasma membrane. 2) The TSHR has a unique 50 amino acid region in its ectodomain which undergoes proteolytic cleavage. The proteolytic cleavage of the ECD may have weakened the interaction by orienting the ECD differently between the ectodomain partners making it susceptible to point mutational changes. The recently solved crystal structure of the TSHR complexed with M22 Fab showed no evidence of dimer formation [26] as opposed to the weak dimerization observed between the FSH-FSHR structures [25]. If these differences are due to packing of the crystals or the inhibition of dimerization by the Fab fragment of receptor antibody [16] remains to be explored further.

To examine if residue Y116 had any role on receptor stability we studied receptor degradation in the presence and absence of this mutation. As a control we included another residue, a leucine I117, adjacent to Y116 and which is also on the outer loop of LLR3. The presence of the Y116 mutation induced degradation of the receptor but the degradation was not strong enough to cause a change in signal transduction i.e cAMP response although it is not know if this would affect other receptor functions such as internalization and down regulation.

In conclusion, the present study demonstrated that the TSHR ectodomain is capable of forming higher order multimers even in the absence of the transmembrane region. Furthermore, Y116, a conserved tyrosine residue on LRR3 of TSHR, was a major contributor to the ectodomain interactions and had a role in the stabilization of the receptor. Nevertheless, since the Y116 mutation did not abrogate multimerization in the full length receptor, the transmembrane region interactions must be the more dominant force.

## Materials and Methods

### Epitopes Tagging of GPI-TSHR ECD DNA

In order to perform co-immunoprecipitation and in vivo FRET studies using a GPI linked TSH receptor ectodomain construct (GPI-TSHR-ecd), we inserted HA or Myc epitope tags on the N -terminus of this construct. The epitopes were introduced immediately after the signal peptide sequence in the GPI-TSHR-ecd cDNA using the method of inverse PCR mutagenesis [41]. After ligation, the PCR product was Dpn I enzyme digested to remove the parent methylated DNA and transformed into INFα' bacteria (Invitrogen) and the positive clones were picked by PCR with HA (human influenza hemagglutinin protein) or Myc (c-myc is a family of nuclear protein found in human tumors) as forward primers and a TSHR gene specific reverse primer. The positive clones were sequence verified for insertion and correct orientation of the epitope tag.

### Cell Culture and Transfection

TSHR ectodomain construct (residues 1–412) linked to GPI (GPI-TSHR-ecd) provided by Dr. A.P Johnstone, St. George's Hospital Medical School, UK, [42] was transfected into Chinese Hamster Ovary (CHO) cells to make a stable line, named G13 cells. These cells were maintained in Hams-F12 with 10% FBS, 100 units of penicillin/streptomycin and 600 ug/ml of G418. For transient transfection experiments, we used human embryonic kidney (HEK) 293 cells obtained from the ATCC. These cells were grown in DMEM with 10% FBS, 100units of penicillin/streptomycin until transfection. The cells were seeded to obtain 80–90% confluence prior to transfection and different doses of DNA were transfected using Lipofectamine 2000 (Invitrogen) as per the manufactures protocol. For FRET studies the cells were seeded in 0.1% collagen precoated Delta T dishes (0.3×10^6^/dish) and transfected with the respective donor and acceptor tagged constructs single or double transfected. 48 hrs post-transfection the cells were stained using either anti-HA conjugated to FITC or anti-Myc conjugated to Cy3 (1∶100 dilution).

### Staining & Expression

The expression of the tagged constructs on the surface of transfected HEK 293 was confirmed by staining unfixed cells with primary antibody (anti-HA MAb and anti-Myc Mab) at 1 µg/10^6^ cells. This was followed by secondary anti mouse (Fab') conjugated to phycoerythrin (PE) (Jacksonimmuno Research Inc) at 1∶200 dilution. Non-transfected and vector alone transfected cells stained with primary and secondary antibodies and secondary antibody alone were used as controls in the assay. The stained cells were analyzed under the FL2/PE channel (575 nm) on a Beckman Coulter Cell Lab Quanta SC flow cytometer.

### Immunostaining and FRET

Transiently transfected cells on Delta T dishes were fixed with 4% paraformaldehyde for 30 minutes at RT and then stained with anti HA conjugated to FITC or anti Myc conjugated to Cy3 in PBS containing 2%FBS for 1 hr. The cells were thoroughly washed with PBS and mounted using Vectashield. Individual dishes with anti-HA FITC as donor (D), anti-Myc Cy3 as acceptor (A) and cells co-transfected with HA and Myc tagged DNA and stained with anti HA FITC plus anti Myc Cy3 as double labeled cells (DA) were used for sensitized emission FRET studies on a Nikon TE 2000-S microscope consisting of emission and excitation FRET filter wheels controlled by In-Vivo software (Media Cybernetics Inc). The four sets of images that were acquired from each stained dish were as follows: 1) Donor excitation-Donor Emission (Dex-Dem), 2) Acceptor excitation-Acceptor emission (Aex-Aem), 3) Donor excitation-Acceptor emission (Dex-Aem) and 4) Acceptor excitation- Donor emission (Aex-Dem). A minimum of 5 cell images from each of the above stained dishes was acquired for FRET efficiency (%) calculations. Using the pFRET software, background subtraction and donor spectral bleed through (DBST) and acceptor spectral bleed through (ABST) were subtracted from the acquired images to give the corrected FRET image and efficiency calculations. The wild type receptor tagged with either GFP or Myc at its carboxyl end were used in the same configuration as mentioned above as positive controls because these receptor constructs have been shown to homodimerize by our previous FRET studies [14], [16].

### TSH Treatment and cAMP Generation

For dissociation studies and cAMP generation, bovine TSH was used (Sigma). The cells were treated in complete medium with 0 and 10^3^ µU/ml of TSH for 30 minutes at 37C for dissociation and for cAMP estimation cells were treated with 0, 10,10^2^,10^3^, and 10^5^ µU/ml of TSH for 1 hr at 37°C. For membrane preparations, the cells were washed immediately with ice cold PBS and chilled on ice for 15 minutes and then prepared by the digitonin method as described earlier [16].For cAMP assays the cells were lysed and an EIA performed as per the instructions of the manufacturer (GE Healthcare Amersham cAMP Biotrak EIA system Cat #RPN225).

### Point Mutations of ECD

Point mutations of Y116 and C176 were introduced by using the Phusion site-directed mutagenesis kit (BioLabs). The protocol from the manufacturer was followed in order to introduce the mutation into the cDNA template of human TSHR. The primer sequences used for introducing the Y116 mutation were 1) Forward primer: 5′-AACTTAACTG-CCATAGACCCTGAT-3′2) Reverse primer: 5′ATCAGGGTCTATGGCA GTTAAGTT-3′ and for Cystein 176 mutation 1) Forward primer: 5′-AGTAATGAAACCTTGACACTGAAG-3′ and 2) Reverse primer: 5′-ACTTAGTCCCTGAAAAGCATTCAC-3′. All mutations were sequence verified and expressed transiently in HEK293 cells to verify surface expression of the mutated protein. A similar approach was used to introduce a conserved point mutation of residue alanine 111 as a nonspecific mutation on the outer loop of LLR3.

### Total Membrane Preparation

CHO-GPI-TSHR-ecd (G13) stable cells were seeded at a density of 5×10^6^ cells per 100 mm dish and allowed to grow to a density of 100% in Ham F12 medium with 10% FBS and antibiotics prior to treatment with TSH. For transient transfection of HEK 293 cells, the cells were seeded at a density of 7-8×10^6^/100 mm dish and allowed to grow overnight to a density of 80–90% before transfecting then with HA-GPI-TSHR-ecd or Myc-GPI-TSHR-ecd or both DNAs. Membranes from test and control cells were prepared as previously described using the digitonin method [16].

### Co-Immunoprecipitation and Immunoblotting

Membranes prepared from transiently transfected cells of HA-GPI-TSHR-ecd, Myc-GPI-TSHR-ecd, double transfected cells such as HA-GPI-TSHR-ecd+Myc-GPI-TSHR-ecd and Y116 mutation HA-GPI-TSHR-ecd + Myc-GPI-TSHR-ecd and C176 mutation HA-GPI-TSHR-ecd+Myc-GPI-TSHR-ecd were immunoprecipitated with anti-myc (9E10 MAb). Briefly, fractions were precleared with protein-A agarose beads for 30 minutes at 4°C. The precleared supernatant was collected into fresh tubes and precipitated by the addition of 1 µg/ml of monoclonal Myc antibody for 3 hr at 4°C. This was followed by a pull down of the immune complex with protein-A beads. The immunoprecipitates were then solubilized with 100 mM of DTT for 45 minutes at 50°C and run on a 12% SDS-PAGE gel and electro-blotted onto PVDF membranes. Membranes were blocked with 5% dried skimmed milk in TBS (Tris Borate Saline) containing 0.05%Tween-20 (TBST) and then probed with 2 µg/ml monoclonal anti-HA for 1 hr at room temperature. Washed membranes were then incubated with 1∶5000 of secondary antibody (anti-mouse HRP) for 1 hr at room temperature. After final washing, bound secondary antibodies were visualized using enhanced chemiluminescence (Super Signal ECL, Pierce, and IL).
